# “Let’s get the best quality research we can”: public awareness and acceptance of consent to use existing data in health research: a systematic review and qualitative study

**DOI:** 10.1186/1471-2288-13-72

**Published:** 2013-06-04

**Authors:** Elizabeth M Hill, Emma L Turner, Richard M Martin, Jenny L Donovan

**Affiliations:** 1 School of Social and Community Medicine, University of Bristol, Canynge Hall, 39 Whatley Road, Bristol, BS8 2PS, UK

**Keywords:** Medical record, Informed consent, Selection bias, Secondary research, Confidentiality

## Abstract

**Background:**

Opt-in consent is usually required for research, but is known to introduce selection bias. This is a particular problem for large scale epidemiological studies using only pre-collected health data. Most previous studies have shown that members of the public value opt-in consent and can perceive research without consent as an invasion of privacy. Past research has suggested that people are generally unaware of research processes and existing safeguards, and that education may increase the acceptability of research without prior informed consent, but this recommendation has not been formally evaluated. Our objectives were to determine the range of public opinion about the use of existing medical data for research and to explore views about consent to a secondary review of medical records for research. We also investigated the effect of the provision of detailed information about the potential effect of selection bias on public acceptability of the use of data for research.

**Methods:**

We carried out a systematic review of existing literature on public attitudes to secondary use of existing health records identified by searching PubMed (1966-present), Embase (1974-present) and reference lists of identified studies to provide a general overview, followed by a qualitative focus group study with 19 older men recruited from rural and suburban primary care practices in the UK to explore key issues in detail.

**Results:**

The systematic review identified twenty-seven relevant papers and the findings suggested that males and older people were more likely to consent to a review of their medical data. Many studies noted participants’ lack of knowledge about research processes and existing safeguards and this was reflected in the focus groups. Focus group participants became more accepting of the use of pre-collected medical data without consent after being given information about selection bias and research processes. All participants were keen to contribute to NHS-related research but some were concerned about data-sharing for commercial gain and the potential misuse of information.

**Conclusions:**

Increasing public education about research and specific targeted information provision could promote trust in research processes and safeguards, which in turn could increase the acceptability of research without specific consent where the need for consent would lead to biased findings and impede research necessary to improve public health.

## Background

Secondary use of health data is common in epidemiological research and reviews of medical records can be of great benefit in large-scale public health studies due to the wealth of pre-collected data available. The British government plans to make de-identified National Health Service (NHS) data readily available for re-use by the private sector, unless patients actively opt out [1]. Currently in the UK informed consent must be sought from individuals for any use of their identifiable data, including when conducting a secondary review of a medical record [2]. In exceptional circumstances approval can be sought to waive informed consent, but in practice this can be difficult to obtain [3-5].

Researchers are concerned about selection bias (or “consent” or “participation” bias) arising from seeking consent, where systematic differences arise between those who consent and those who do not. The detrimental effect of selection bias on the validity of data has been shown by a number of studies [6-10], although not all [11]. As there is no effect on the patient or their care from this type of secondary research, some researchers argue that consent for a review of the patient’s record is unnecessary [4,12-14], and that similar audit-based reviews of records are routinely undertaken by clinicians, without requiring separate informed consent [15,16]. It is claimed that NHS medical records are a comprehensive resource funded by public money and therefore should be used to further research for public benefit [1,13,16,17], and that the cost of consenting is too high and practical obstacles too great [18-20]. Researchers often note that many participants cannot be contacted because their clinician denies access, or they do not respond, while few potential participants actively refuse to take part [4,7,8,19].

Although researchers may wish for easier access to medical records to reduce potential bias and the cost of the consent process, public opinion may not be so permissive. Members of the public hold a wide range of views about the necessity of consent for a review of their medical records [21-23]. Some would like to be offered an opportunity to consent for each use of their data; for others one-off consent would be acceptable to cover all future research, and some are happy for such research to go ahead without informed consent. A number of studies have suggested that members of the public are more willing to waive consent when they understand the issues involved in carrying out such studies. Two large-scale UK surveys concluded that the public has low awareness of how their medical data are used, and if they are informed about what research entails they are generally more positive about it [22,23]. In other studies where the public were asked about consent for secondary uses of medical data, participants were found to be poorly informed about research processes and existing safeguards, and education was called for to engage participants and increase the acceptability of such research without consent [24-29]. Engaging the public and increasing awareness about research using medical data was a key recommendation in a UK report on the use of personal medical data for public good [30].

This study included a systematic literature review and focus group study. The literature review provided an overview of the existing evidence and enabled the findings from the focus group to be set in a broader context. The focus group study was part of the ongoing CAP (Cluster randomised triAl of testing for Prostate cancer) trial, which is evaluating the population effectiveness of a single prostate specific antigen (PSA) test for prostate cancer [31]. In the focus groups, we elicited views about consent for a review of existing medical records in general. Participants were provided with information about research processes and selection bias so that we could investigate whether an understanding of selection bias would alter views about the necessity of consent for a review of medical records.

## Methods

### Systematic review

We searched for any qualitative, mixed method or survey design study that mentioned reasons or characteristics behind different consent preferences for secondary review of pre-collected health data. Medical data could be held in primary or secondary care, and be electronic or paper-based. Studies requesting consent to any intervention were not included; studies concerned with consent for use of secondary data only were included. There were no restrictions on publication date or publication type in the search. The search terms were applied in English only, however, non-English language papers were not excluded. Conference abstracts were included.

#### Search strategy

Studies were identified by searching electronic databases, and by reviewing the reference lists of included articles for any further studies that met the inclusion criteria. We searched PubMed (including Medline) (1966-16^th^ January 2012) and Embase (1974-16^th^ January 2012).

We used the following electronic search strategy for all database searches:

((consent OR authorization OR authorisation) AND bias [title + abstract only]) OR ((confidentiality OR privacy) AND (medical record OR health record OR health information) [title + abstract only]).

#### Study selection

**Figure 1:**
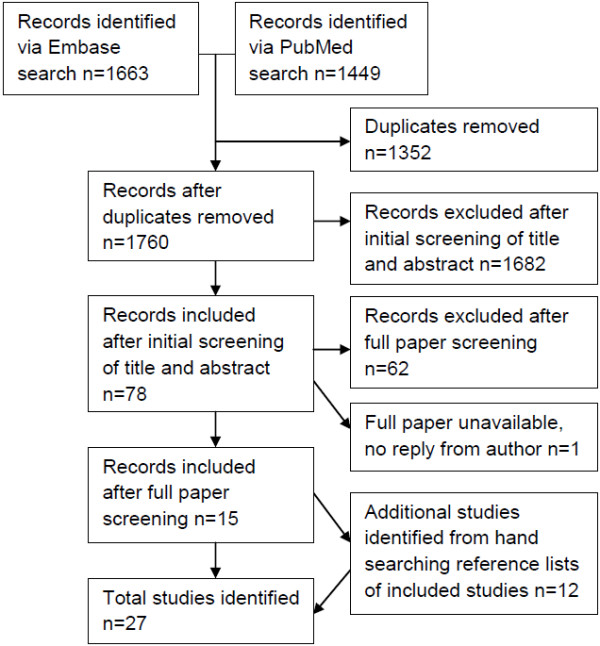
Flow diagram of included studies.

The flow diagram in Figure 1 shows the papers retrieved and excluded at each stage of the systematic review. Titles and abstracts of all articles were reviewed by EH. Any articles potentially fitting the criteria, or where this was unclear from the title and abstract, were retrieved and the full text articles were reviewed. Data from papers included in the full review were abstracted to a standardised form. Data included type of study (e.g. survey, focus group), sample size, participant characteristics including age, gender and any health condition or status. The main findings from each paper were summarised. As this review sought both qualitative and quantitative research, a summary statistical analysis was not performed.

### Focus groups

We used qualitative methods to explore the views and opinions of members of the public about data from a review of their hospital medical records being extracted and used for research purposes. The focus group methodology was chosen as this allowed exploration of the opinions of participants in general before presenting them with information about selection bias and noting the ways in which their understanding and opinions changed in relation to the information provided. Focus groups are considered especially useful where the issues under discussion are new to people, allowing exploration of ideas they may not have considered before [32].

#### Participants

Focus group participants were men potentially eligible for the CAP study, aged between 50 and 69 years, but who were not recruited to the study because of their location away from the study areas. Participants for the focus groups were identified at random from the lists of two primary care practices. To increase the generalisability of focus groups we approached primary care practices located within an area of some deprivation (higher deprivation quintile) and a more affluent area (lower deprivation quintile), as ranked by the UK Index of Multiple Deprivation [33]. Patients who were considered to have terminal illness or who the General Practitioner (GP) deemed unsuitable to participate in the focus groups for other reasons, for example learning difficulties, were excluded.

#### Methods

The focus group study received research ethics committee approval from Dorset Research Ethics Committee (ref: 07/H0201/108). Participants meeting the inclusion criteria were sent an initial invitation letter outlining the study and requesting they return a reply slip if they were interested in receiving further information. Interested men were sent an information sheet, consent form and short questionnaire, which asked for information about their general health using the 12-item Short Form Health Survey (SF-12) [34], and demographic information to allow us to balance the participants in each group according to age and general health status. Men indicated whether they had worked for the NHS or had ever taken part in health research so we could gauge the extent of their prior knowledge about health research.

A total of 19 participants attended three focus groups, with between 5 and 9 in each group. The number of focus groups was limited due to time constraints. All groups were facilitated by EH, with a note taker and second researcher present, and followed the same topic guide, each lasting approximately 90 minutes. Following initial questions about the contents of hospital medical records, intended to relax the participants and initiate discussion, the men were then asked how they would react to a letter asking them to take part in a study that wished to review their hospital records. They were asked if they would be willing to let a researcher have access to their records for research purposes. We provided information about different methods of consent: opt-in, informed consent and opt-out, or presumed consent, and asked whether they would approve of their records being reviewed without prior consent being sought.

In order to ensure that participants understood the processes and some of the issues faced when undertaking such research, a presentation was then given about possible selection bias arising from having to obtain informed consent; that is, by obtaining consent, certain groups of people would be more or less likely to agree to participate, which might alter the findings. Information was presented verbally along with text and diagrams, and was given in general and then in the context of a real-life scenario. There were opportunities for questions throughout and we checked understanding of selection bias by monitoring their response, and providing further clarification if necessary.

The participants were then asked whether the possibility of selection bias changed their opinion about a review of their records without informed consent. To conclude, we discussed whether any safeguards could be put in place that would reduce any individual’s requirement or preference for informed consent. Throughout, participants were requested not to talk about their own medical conditions or experiences.

#### Analysis

All focus groups were audio recorded with each participant’s permission, transcribed verbatim and anonymised. At least two researchers (EH and JD) undertook iterative thematic analysis of the transcripts using the method of constant comparison to generate a list of themes. Themes were compared within and across focus groups and participants, and the coding and themes were discussed until all researchers (EH, JD, RM and ET) agreed with the final list.

## Results

### Systematic review

The flow diagram in Figure 1 shows the papers retrieved and excluded at each stage of the systematic review.

Seventy-eight papers were identified as potentially relevant from the title and abstract of the 1760 unique papers found by the literature search. Of these, 68 were excluded on review of the full article. One potentially relevant conference abstract was also identified and the author was contacted for further information but did not reply; this abstract was excluded as it did not contain enough information to meet the inclusion criteria. After full review, 15 papers met the review criteria. Hand search of the reference lists of these 15 papers, and those papers identified from the reference lists, produced another 12 papers. The resulting 27 papers included three papers using qualitative methods, 12 surveys and two systematic reviews of surveys that contained information on the differences between consenters and non-consenters, and 10 papers using mixed methods. The characteristics of the included studies are shown in additional files for both quantitative aspects (Additional file 1a, studies reporting response rates; and Additional file 1b, studies reporting perspectives of respondents) and qualitative aspects (Additional file 2).

Of the twenty-seven included studies, nine were conducted in the USA [28,35-42] with one of these featuring US veterans only [42]. Six studies were carried out in the UK [11,22,23,43-45], one in Eire [46] and one in New Zealand [11]. Eight studies originated from Canada [21,24-27,47-49], with two research projects being reported by two papers each [25,47], and [26,27]. Of the two review papers, one sought only studies from the UK [10], while the other included papers from Canada, UK, USA, Ireland, Japan, Taiwan, and Australia [6].

#### Characteristics of consenters and non-consenters: survey results

Of the twelve quantitative surveys looking at differences between those who consented to a review of their medical records or not, two reported no significant differences between consenters and non-consenters [11,50], seven papers reported that males were more likely to consent than females [24,36,37,40,41,43,48], four reported older respondents more likely to consent [36,37,40,43], while three noted that those with less sensitive or stigmatising information were more likely to consent [24,37,38]. The unemployed [24] or those not paying for their healthcare in the US were more likely to consent [38]. Those in poorer health [40] and people with cancer were more likely to consent than the general public [35]. One online survey of the general public found the reverse: that those younger, in better health and of white ethnicity were more likely to consent [39].

The systematic review of seven UK surveys found that overall those with the symptom under investigation were most likely to consent and consent rates fell in the over 50 age groups, especially for women [10]; however, another systematic review found no clear patterns across 17 international studies on any of variables age, sex, income, education or health status [6].

Patterns of consent were similar in the quantitative aspects of the six mixed methods papers that reported differences in consent rates between groups: male gender [21,22,45], older age [23,45,46], having a less sensitive condition [22], being of non-white ethnicity [22], long term disability [23] and having breast cancer or sickle cell disease (versus other conditions such as cystic fibrosis or colon cancer [28]) were factors associated with being more likely to consent. Participants rating themselves as having a greater knowledge of the NHS were more likely to consent in one study [45]. Higher socioeconomic status was associated with higher consent rates in two studies [23,45], although another study questioning patients with various conditions including cancer and diabetes found those on a lower income more likely to consent [28].

#### Themes arising from previous qualitative research

Three papers were identified reporting qualitative results only. There were also ten mixed methods papers that included a qualitative aspect. The papers focused on five major themes. The key findings are listed below:

Eleven of the 13 studies noted the lack of current knowledge that many participants had about how their medical data may be used for research and the existing safeguards to protect their data [22,23,25-27,42,44-47,49]. There were widespread calls from both the patients and researchers for increased public education about research processes and safeguards.

Participants were reported as recognising the benefit of research for the population in nine of the studies [23,25-27,42,44-47]. The time and effort involved in obtaining consent was often balanced against the public benefit of the research during discussions.

In ten of the studies, participants wished to be informed about how their data were being used and by whom [22,23,25-27,42,44-47]. Information about the user of the data was seen as more important than the intended use in determining whether to offer consent in some studies [22,45], while others noted that the planned use of the data was an important determinant of whether participants would consent to its use [26,42,47].

Different consent models were discussed in ten papers [22,23,25,26,42,44-47,49]. There was no consensus on a preferred model either within or across studies, although participants often considered the balance of obtaining consent against the public benefit incurred by unrestricted research. Despite this recognition, many participants maintained that informed consent should always be sought, out of respect for the individual.

All 13 studies mentioned areas of concern held by participants about data sharing. Data and database security [22,28,46,49], and whether the data were anonymous was a concern, with participants being less restrictive when data were anonymous or unidentifiable [22,26,28]. There was apprehension in many studies that data would be sold for commercial profit, and this was generally seen as less acceptable, commanding a higher requirement for informed consent [21,23,25-27,42,44,45,47]. Release of data to insurance companies or pharmaceutical companies was often mentioned as a concern, again leading to a more restrictive consent requirement [23,26,27,42,44,45,47].

### Focus groups

**Figure 2:**
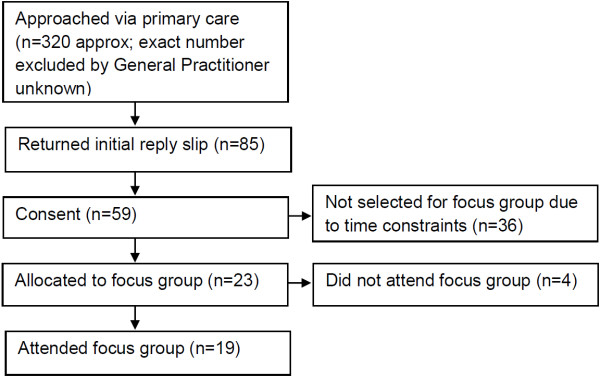
Focus group response figures.

Figure 2 shows the focus group response figures. Approximately 320 men were invited (exact number of men excluded by GP unknown), 85 were sent information about the study at their request and 59 consented to take part. Twenty-three men were scheduled to take part in a focus group, with four of these failing to attend. Nineteen men (mean age 61 years) took part in our focus groups; age, occupational and health status were balanced across groups (Table 1). The characteristics of the men who consented to be involved and returned a questionnaire (n = 58) were very similar to those who attended the groups.

**Table 1:** Sociodemographic characteristics of focus group participants (n=19)

	**n**	**%**
Mean age in years (SD)	61 (4.84)	
Age range	54-69	
Marital status		
Married / living as couple	16	84
Single	1	5
Divorced	2	11
Ethnicity		
White British	18	95
White other	1	5
Employment status		
Employed	11	58
Retired	5	26
Unemployed seeking work	1	5
Unemployed due to illness or disability	2	11
Mean SF-12 score*		
Physical health component (score*)	(52.06)	
Mental health component (score*)	(51.32)	

*SD* standard deviation.

*Range from 0 to 100, with scores below 50 indicating poorer than average health.

One participant reported having formerly worked for the NHS (not in a clinical role). No participants reported taking part in any health research previously; however, one participant had some knowledge of health research having sat on a local research ethics committee.

#### Consent for medical record review

All participants accepted the need for research and understood that this was an important aspect of the NHS. A few expressed surprise that this sort of research was not happening as a matter of course. All would consent to a review of their medical records if asked:

“Definitely if it was going to benefit somebody else then yeah.” (Participant 2, group 2)

When asked about the use of records without their prior informed consent, opinions were split equally between those that saw the “greater good” of public benefit and those who thought it was courtesy that they were informed:

“I’m saying yes because I think there’s a greater good.” (Participant 1, group 2)

“I think it’s just etiquette to ask people to do such things.” (Participant 5, group 3)

#### Understanding of selection bias

Following presentation of information about selection bias and discussion of issues surrounding the consent process, participants could understand the difficulties faced by researchers. They recognised the increased cost and time that the process took and how a low response rate might bias the findings:

“Well I’d sympathise with the researcher and I think if you’re setting yourself up to do research which is going to be skewed by the nature of consent then let’s try and avoid the issue of going for consent. Let’s get the best quality research we can do.” (Participant 1, group 2)

We asked if knowing this changed their opinion on the use of their medical records without their prior consent. Many were already positive about the use of records without their prior permission and clarified this, but for those who were more reluctant in the first instance, their opinion did change. However, when asked, a few men still preferred informed consent. This was more to do with an interest or curiosity in the kind of research that they were contributing to. Although they would like to be informed, participants were still likely to consent but felt the need to know details of the research:

“My own personal opinion, as much as I understand the maths of it, is I would still like to be asked.” (Participant 8, group 3)

Among those who preferred to be informed about research, opt-out consent was considered to be acceptable, as it satisfied their curiosity and offered a chance for refusal, with less of an impact on the validity of the results.

#### Safeguards

We asked if there were any safeguards that could be put into place that would make the men more likely to accept the scenario of no prior consent. Common safeguards suggested were anonymisation and data encryption:

“I think I’d be happy as long it is stays within the area, it didn’t find its way into a laptop, and it was all encrypted.” (Participant 1, group 1)

A minority of participants said there could be no safeguards that would make them happy with the no consent scenario:

“I don’t want any safeguards, end of story, so I want prior consultation.” (Participant 5, group 3)

Interestingly, no participants spontaneously mentioned ethics committees, NHS research governance procedures or legislation when considering potential safeguards. Throughout the discussion many questions were asked about how research was carried out, suggesting that participants were unaware of how their data could currently be used, and needed to be able to trust that their data were secure:

“How do we know that you just don’t go to the hospital and say “can I have a look at these records” and we don’t know anything about it?” (Participant 8, group 3)

#### Potential misuse of information

The fear of data being misused by companies for their own gain was very apparent in all groups. Concerns were mainly around insurance companies obtaining health information which may affect their premiums or cover, or companies using the information for targeted advertising:

“What I don’t like is any information being passed on to a third party, for promotion purposes. Say you’ve got a particular problem then it goes to a drugs supplier or something like that, that I would object to.” (Participant 4, group 1)

Despite the information collected from medical records being anonymised, the men had the same concerns over their personal information being disclosed inappropriately by the researchers as by commercial companies:

“I think we can forget security because let’s face it, it isn’t there anymore so if you don’t want it to be given out then you say so…” (Participant 2, group 2)

Although some men seemed resigned to the fact that there was a lack of security, they were still willing to consent and would accept the security risk as part of the process.

#### Acceptable & unacceptable types of research

In all groups there was discussion around acceptable and unacceptable types of research. This dichotomy was based on who profited from the research, rather than the study design or ethical aspects. Research undertaken by the NHS was seen as acceptable and for public good, whereas pharmaceutical companies who gained financially from the altruistic sharing of records were seen as less acceptable:

“If there was a large commercial company… [that] had free and easy access to people’s medical records I don’t think that would be right. It would further their research into the particular drug or treatment, but it’d also further their profits that would be wrong. But if it was for medical research for everybody then that would be different.” (Participant 6, group 3)

“Financial gain comes into it then so why should you then let them look at your records? They’re going to gain out of it and you’re not…” (Participant 2, group 2)

University researchers were considered to be somewhere in the middle, and it was the funder of the research and their financial gain that was considered when making a judgement about the acceptability of the research:

“The question would be who are the researchers working for? Are they researching for medical companies, or universities who are attached to medical companies and getting funded [by them]?” (Participant 8, group 3).

There was an apparent dichotomy between acceptable research, seen as being undertaken for the public good, and less acceptable research, for commercial gain.

#### The impact of the provision of information

Men became more accepting of research without specific individual consent following the provision of information about bias and research processes, for example the time and cost of obtaining consent:

“If you’re putting money into a charity for them to research something, you want that research done, not for it to be spent on the administration to enable the research to be done.” (Participant 5, group 2)

Where concerns remained in the minority, on the whole these related to the potential misuse of their personal data, or a wish to be informed about the use of their data. Participants questioned how the processes would work, and their need to trust that the data would be secure was implicit:

“How would it happen, how would you do it, how would you keep it secure?” (Participant 3, group 1)

## Discussion

The systematic review identified twenty-seven relevant studies. From the quantitative literature, males and those who were older seemed more likely to consent to a review of their medical records, although this was not confirmed in a meta analysis of 17 international studies [6]. Similar themes arose in the qualitative studies. Participants recognised the benefit of sharing their records for research, but the majority wanted to know how their data were being used. The systematic review found that participants shared many of the same concerns about disclosing their health data, although the focus on certain issues reflected the context of each study. Concerns over data security, data being used by insurance or pharmaceutical companies and data being sold for commercial gain were common. The UK government’s plans to allow commercial access to NHS data [1] may be unacceptable to the public unless a benefit for the majority can be demonstrated. Nearly all the identified studies noted that participants were ill-informed about current practices and use of their health data and that educating the public may increase the acceptability of the use of data for research. Despite the research being carried out in a number of countries, each with different health care systems and governing laws, the themes were very similar, suggesting that there are common issues which need to be addressed if increasing numbers of members of the public are to agree to a review of their existing medical data.

Our focus group research explored men’s views about consent for a review of medical records in the context of selection bias and the findings reflected the themes arising from the systematic review. All the men involved agreed that they would consent to a review of their records when asked. However, opinion was split when informed consent was not to be sought for such research. Some were happy to be able to help the “greater good”, yet others, while understanding this opinion, felt that it was only courteous to be informed about the research and that the data should not be taken without the patient’s knowledge. This accords with previous research that has shown a wide range of public opinion regarding consent to review of medical records [21-23]. Following discussion about selection bias, participants’ views about research without consent became more favourable, with some men changing their opinion and no longer stating the need for specific informed consent. However a small minority remained adamant that they always want an opportunity to consent or at least to have an opt-out consent option. The increasing acceptance following information provision seen in the focus groups was in contrast to two similar studies (reported in three papers) that provided information and research scenarios to aid understanding and found that although individual opinions altered throughout the course of the dialogue, aggregated opinion showed little change [25,42,47]. Focus on selection bias specifically could perhaps influence the opinions of the participants to a greater extent than more general consent scenarios and future research should investigate whether this is seen in other demographic groups, and the best way to present this information to ensure a wide public understanding.

When safeguards were mentioned by the facilitator, the participants did not seem to value them as tangible and asked many questions. There was a lack of knowledge about current safeguards and there was no spontaneous mention of ethical approval, hospital research governance procedures or the contractual obligations of the researchers by the participants. In one group there were questions about data protection laws, but on the whole participants failed to recognise these existing safeguards. The discussion and questions generated showed that the participants were interested in this and wanted a greater understanding of the processes involved. Previous research has also suggested that the public feel poorly informed and would like more information about how research is carried out [22-29]; eleven of the thirteen qualitative studies identified in this systematic review also noted their participants’ lack of knowledge.

There were concerns from these focus group participants about possible misuses of health information such as passing on health status information to insurance companies or to target advertising for certain treatments. Older men in another study also feared that records could be shared with outside agencies [42], and these concerns were highlighted in other research [23,51]. In the focus groups, the dichotomy between acceptable and less acceptable types of research was based on who profited from the research, rather than the study design or ethical aspects, with NHS research for the benefit of the public seen as an example of good research and pharmaceutical companies acting to gain financially seen as less acceptable. Scepticism about research for commercial profit was a common theme and a number of studies noted that participants wanted more restrictions on their data where commercial profit could be made from their information [21-23,25-27,29,50]. If altruistic sharing of data resulted in profits that did not benefit the NHS, participants would have strong reservations about allowing access to their records. Public education about current research legislation and data security measures is needed to allay misconceptions about the use and safety of patient data, and this will be of paramount importance if the proposed access to data by private industry is to be acceptable.

Many similar themes have been found in the related area of secondary use of tissue samples for research. A review of 30 studies reporting views on consent for research using biological samples [52] found a high level of willingness to donate leftover samples for research (83-99 %), with marginally less support for commercial rather than academic research. Nine of the studies noted that people would like information on how their samples were used. A recent review of 18 qualitative research papers also noted comparable themes to those reported here [53]. Reasons given for allowing excess tissue to be used for research were for both individual gain and for public good, but most patients agreed that tissue should not be used without the patient’s consent. Trust in research institutions that data would be secure and tissue not exploited was a common theme, with similar concerns about commercial access to samples as seen in our research.

This study was the first to obtain qualitative opinions about medical record review both before and after provision of detailed information about selection bias and research processes. By providing information about the problems of selection bias, we were able to explore how opinions changed in the context of this knowledge. We were also able to answer the many questions that arose, which helped to increase understanding of research amongst our participants. Previous work has noted that during interviews about consent for medical record reviews participants were still formulating their thoughts and did not have mature opinions [26]. The focus group format allowed our participants to develop their opinions in an area that was new to them, gaining insight from the views of others [32]. Our focus groups concentrated on the provision of specific consent for each project, however it is possible that participants may have found broad consent for a number of similar projects to be satisfactory. Opt-out consent rather than opt-in consent may also be acceptable and future research should explore this further. Women and younger males were not included in our focus groups, and all participants described themselves as of white ethnicity, so these are limitations. The systematic review identified males and older people as more likely to consent to a review of their medical records and, as is true with all research, those who agree to take part are often those who are more positive about research. Therefore the opinions generated by the focus group may be more accepting of such research without consent than the general population. Despite this, our focus groups found that there were still a number of concerns to be allayed about the research process and that information provision may be useful to reduce concerns in all groups, not just in those who are positive about research in general.

## Conclusions

The majority of men in our focus groups were happy for medical record reviews to go ahead without consent, and even more so when their understandings of selection bias and appropriate safeguards were enhanced. Increasing public education about research is likely to benefit research, as a greater understanding of the safeguards and legislation that governs research should increase public trust and reduce misconceptions. This could improve consent rates or, as found here, the acceptability of research without informed consent for “the greater good” of the National Health Service.

## Competing interests

The authors declare that they have no competing interests.

## Authors’ contributions

EH, ET, RM and JD conceived and designed the study. EH performed the systematic review and designed the focus group guide and facilitated all groups with JD, ET or RM also present. EH and JD analysed the focus group data. EH drafted the manuscript and all authors reviewed and approved the final manuscript.

## Pre-publication history

The pre-publication history for this paper can be accessed here:

http://www.biomedcentral.com/1471-2288/13/72/prepub

## Supplementary Material

Additional file 1 Characteristics of included studies – Quantitative aspects (1a, studies reporting response rates; 1b, studies reporting perspectives of respondents). Click here for file

Additional file 2 Characteristics of included studies – Qualitative aspects. Click here for file
