# Supplementary material for: “Let’s get the best quality research we can”: public awareness and acceptance of consent to use existing data in health research: a systematic review and qualitative study
Source: BMC Med Res Methodol. 2013 Jun 4;13:72. doi: 10.1186/1471-2288-13-72 (PMC3682867; doi:10.1186/1471-2288-13-72)
Supplement: Additional file 1 — Characteristics of included studies – Quantitative aspects (1a, studies reporting response rates; 1b, studies reporting perspectives of respondents). [file 1471-2288-13-72-S1.pdf]

Additional File 1a: Characteristics of included studies – Quantitative aspects – Response rates

Study	Setting	Participants*	Request	Main results	Most likely to consent
Baker 2000	Survey, UK	5069 primary care patients (2679 with asthma, 2390 with angina) aged 16 or over.	Questionnaire on symptoms with request to collect additional information from medical records.	9.8% of questionnaire responders refused MRR.	No significant difference between consenters and non-consenters for age, sex, severity of symptoms or satisfaction with care.
Bolcic-Jankovic 2007	Survey, USA	2030 previously hospitalised patients aged 18 or over, who agreed to be mailed an authorisation form to allow researchers to review their records.	Telephoned after discharge with request for MRR. Those willing sent one of various forms, allowing analysis of form characteristics that led to higher consent.	50% returned signed form. Requesting social security number on the form had the single greatest negative effect on return.	Males, older (interaction)
Damery 2011	Survey, UK	599 primary care patients with iron-deficient anaemia.	Letter and consent form with request for MRR.	71% returned consent form, 63% consented to MRR, 8% refused (29% did not reply)	Older, males
Dunn 2004	Review, UK surveys	42812 members of the UK public, aged 18 and over, approached for one of 7 general population surveys.	Meta analysis of 7 UK pop surveys on consent for medical record review.	Overall response rate 65%. Consent rate increased from youngest until 40-49 years, then fell continuously for both male and females over age 50. Consent under age 50 was similar in males and females, but over age 50, more males consented than females.	Males, younger, those with symptom under investigation
Jacobsen 1999	Survey, USA	2463 randomly identified patients, aged 20 or over, treated at a primary and tertiary care centre.	Postal request for authorisation for medical record review.	83% responded. Overall refusal rate 3% (21% including non-responders). Higher Charlson score associated with lower authorisation. In general, those with conditions considered more sensitive were more likely to refuse authorisation.	Older, males, without a sensitive condition, those living further from treatment centre
Kho 2009	Review, international surveys	161604 patients from 17 studies (5 UK, 5 USA, 3 Canada, 1 Ireland, 1 Japan, 1 Taiwan, 1 Australia) reporting characteristics of consenters and non-consenters.	Systematic review of prospective observational studies approaching participants for informed consent for MRR.	Across all studies: 67% consented (range 37%-93%). Variations but no clear patterns across studies on any of variables: age, sex, income, education, health.	No clear pattern across studies

Merz 1999	Survey, USA	240 previously hospitalised patients of any age.	Retrospective MRR to determine rate of completion of release of information form held in records, and whether this was related to presence of sensitive information in the record.	17% of records missing the release of information forms. Of those forms filed: 62% signed, 38% not signed. Only factor significantly related to presence of form was admission as emergency (mostly missing). After correcting for point of admission, two correlated factors were related to a lower chance the form was signed: presence of sensitive information and patient paying for their own care.	Those with less sensitive information in record, and those not paying for care
Woolf 2000	Survey, USA	1106 randomly selected patients attending a primary care clinic not for the first time, aged 14 years or over (minors accompanied by guardian).	Question at end of survey completed in waiting room. Request for permission to be contacted by phone or mail and for MRR.	67% consented to MRR and contact, 25% actively denied consent, 8% didn't answer.	Older, males, those in poorer health
Yawn 1998	Survey, USA	All 15997 patients visiting a multispecialty medical centre, including inpatient, outpatient and emergency department visits, all ages.	Every patient asked to sign authorisation for MRR.	4% refused, 91% granted permission, 1% not asked.	Males, those presenting for mental health reasons, eye care, trauma or gynaecology

* for survey data, the number reported is the number approached unless otherwise stated
FG = focus group
MRR = medical record review

Additional File 1b: Characteristics of included studies – Quantitative aspects – Perspectives of respondents

Study	Setting	Participants*	Request	Main results	Most likely to consent
Beckjord 2011	Survey, USA	8411 respondents to online survey, recruited via Livestrong plus respondents to the Health Information National Trends Survey (HINTS) performed by the National Cancer Institute; 4092 respondents to telephone interview and 3582 returning postal questionnaire.	Questions on willingness to share electronic health data for research.	From the Livestrong sample, 70% of those living with cancer as a chronic illness strongly agreed that researchers should be allowed access to anonymous records, with around 60% of those treated for cancer and 56% of those with no personal history of cancer willing to share their records.<br>From the general population HINTS survey, 32% strongly agreed that researchers could access their anonymous data.	Those with cancer more likely than those with no personal history of caner or the general population

Buckley 2011	Mixed methods, Ireland	Survey: 3883 adult members of Irish public identified via postal service database to be nationally representative of age, SES and region, aged 18-85 years.	Questionnaire on consent preferences for example scenarios.	41% responded. 10% preferred consent for each use of data, 68% endorsed the GP to decide whether to release anonymous data, but preferred to be asked when data identifiable, 22% happy for data to be used without consent. Low understanding of content of records.	Older, retired, completion of primary education
Damschroder 2007	Mixed methods, USA veterans	Survey: 3618 randomly sampled patients from Veterans Affairs (VA) facilities balanced by age, ethnicity and number of clinic visits.	Phone survey on attitudes about research and willingness to share records both before and after deliberation session.	Most inclined to share data with VA researchers (89%), followed by university researchers (75%), a preventative health program at a local hospital (61%) and a drug company for marketing purposes (51%). No change in attitudes post-deliberation.	No demographic comparisons made
Kass 2003	Mixed methods, USA	602 patients in one of 6 equal groups: cystic fibrosis, sickle cell disease, diabetes mellitus, HIV, breast cancer and colon cancer, recruited from outpatient clinics, ongoing research, disease registries and newspaper advertisement.	Interviews by phone or in person. The interview contained both qualitative and quantitative items.	31% agreed that researchers should have access to their data without their permission; 55% disagreed. Those with an income <$20,000 were twice as likely to agree that researchers should use data without permission ($p<.05$). Breast cancer patients had highest agreement rates.	Lower income, and those with sickle cell and breast cancer had highest agreement rates
MacKinnon 2006	Mixed methods, Canada	Reports same study as Willison 2008. 98 members of the public recruited by random digit dialling or via invitation at the end of public opinion survey.	Citizens' dialogue - day-long deliberations, balanced information in advance about 3 consent approaches; Completed questionnaire before and after discussion, rating support for each of 3 scenarios: permission each time, permission not needed but want to be informed, broad consent.	Participants gave broad consent the highest rating, both pre and post the dialogue session, with around 80% supporting this method. Approx 45% supported project by project consent; approx 50% supported assumed consent. There was little change in patterns of support following the dialogue.	No demographic comparisons made

MRC 2007	Mixed methods, UK	Survey: 2106 members of the public, nationally representative selected from a varied range of parliamentary constituencies, aged 15 and over.	Questions on face-to-face omnibus survey administered in the home.	69% say likely or certain to allow their data to be used for research, 25% unlikely or certain not to. Most common reason for not sharing is concern over privacy (28%) with only 1% against medical research. GPs are trusted by 87% to have access, hospital doctors by 59%. Public sector researchers are trusted by 11%, private sector researchers by 4%. 21% would be more inclined to consent if they had information about purpose of research, or knew more about it (17%). 40% of those who were unlikely to give consent said they are more likely or certain to consent if they were given information about confidentiality.	Older (over 55), Higher socio-economic status, those with long term disability
NHS IA 2002	Mixed methods, UK	2087 members of the public representative of the population, aged 15 and over.	Questions on face-to-face omnibus survey including consent preferences, concerns about data use, scenarios in which no consent would be acceptable.	Little awareness of how data used. 28% would trust university researchers with access to their full record, 23% with full access bar some sensitive information. Most preferred consent for each use, including treatment (35%), or not including treatment (30%), 29% found one-off consent acceptable.	Males, ethnic minorities, less sensitive conditions
Page 2006	Survey, Canada	478 participants with AIDS, Multiple Sclerosis (MS) or a mental disorder, identified from relevant community-based organisations (AIDS: all patients in database invited, MS: every third client in database invited, mental disorder: consecutive presenting clients).	Questionnaire about experience with research, consent and privacy.	Overall response 49% (AIDS 21%, Mental disorder 51%, MS 65%). 83% believed consent necessary for identifiable data, falling to 33% if anonymous. 78% were “consent advocates” with no difference between illness groups. Women and those who were employed were more likely to be consent advocates.	Males, unemployed, those with less stigmatizing conditions
Perera 2011	Survey, Canada	490 patients (plus 46 physicians) completing the Health Information Privacy Questionnaire as part of a study of diabetic patients.	Telephone survey with diabetic patients at baseline and end of a study.	22% of patients refused university researchers access to anonymous data (but 67% refused private insurance companies, 45% refused pharmaceutical, 40% refused government, so researchers most favourably viewed). Males more likely to allow use of data than females. No significant changes in views at follow up.	Males

Shickle 2002	Mixed methods, UK	Survey interviews with 3921 members of the public aged 15 years or over and 184 patients and 90 parents of patients recruited in outpatient clinics or inpatient wards.	Comprehensive surveys and qualitative interviews and focus groups, looking at various research scenarios and consent options. Questions on face-to-face omnibus survey including assessment of various scenarios.	For 32% of responses to the scenarios, public happy to allow access to their information. 2% not happy with any scenario, 9% happy with all. The individual requesting the data was the most important factor in determining willingness to allow access to data. For patients there was no association with age or gender and willingness. Patients rating themselves as having more than average knowledge about NHS more likely to be happy to share data.	Public sample: Older, higher SES, males<br><br>Patient sample: higher knowledge about NHS
Westin 2007	Survey, USA	2392 respondents to an online survey of general public, aged 18 and over.	Online survey asking attitudes to research and consent options	50% would be happy with researcher access to de-identified data. 1% happy with for data use with no consent, 8% one-off consent, 19% consent not needed if data anonymous, 38% specific consent each use, 13% wouldn't want health data to be used or to be contacted. No majority consent preference - 20% couldn't choose, despite having had information about potential problems of getting consent.	Younger, white ethnicity, better health
Whiddett 2006	Survey, New Zealand	263 consecutive attending patients at 5 primary care clinics, aged 18 and over (Maori ethnicity over represented).	Questionnaire including attitudes to sharing data and with whom.	Response rate 77%. Approx 45% would definitely share unidentifiable data with researchers, 40% maybe. Approx 15% definitely willing to share identifiable data, 45% maybe. Least willing to share with other organisations (insurance / government). Increased reluctance if information more sensitive. Few consider selves to be well informed about use of health data.	No significant relationship between demographic characteristics and attitude to sharing data
Willison 2003	Mixed methods, Canada	Reports same study as Nair 2004. Survey: 106 patients consecutively approached at doctors office, aged 18 and over.	Questionnaire administered in waiting room about sharing of anonymised data.	91% response rate. 26% found passive notification acceptable, 74% wanted opt-in consent. 57% wanted specific info about the research - goals, benefits, funding (women requested more details than men; 62% vs 46% but not significant).	No demographic comparisons made

Willison 2009	Mixed methods, Canada	Survey: 1780 invited participants, aged 18 and over, with one of 7 stigmatizing conditions: hypertension, diabetes, depression, alcoholism, HIV, breast cancer or lung cancer, recruited from database of those with conditions or via doctors' offices, plus general public reference group recruited by a polling firm.	Telephone or internet survey containing 5 research scenarios with 5 consent options.	403 completed survey. "Disclosure concern" highest among HIV and depression groups, lowest for hypertension, diabetes and lung cancer. Those in HIV group had similar (high) disclosure concern as reference group. Consent profiles similar across all groups including reference group. More concern if data used for marketing or for profit. Predictor of consent choice was individual attitude to medical benefit and disclosure concern, rather than the person's health condition.	Males
Willison 2008	Mixed methods, Canada	Reports same study as MacKinnon 2006. 98 members of the public recruited by random digit dialling or via invitation at the end of public opinion survey.	Citizens' dialogue - day-long deliberations, received workbook in advance, with balanced information about 3 consent approaches; Completed questionnaire before and after discussion, rating support for each of 3 scenarios: permission each time, permission not needed but want to be informed, broad consent.	Participants regarded broad consent the most favourably, both pre and post the dialogue session. There were no significant differences in consent approaches pre and post dialogue.	No demographic comparisons made

* for survey data, the number reported is the number approached unless otherwise stated
FG = focus group
MRR = medical record review
