# Supplementary material for: “Let’s get the best quality research we can”: public awareness and acceptance of consent to use existing data in health research: a systematic review and qualitative study
Source: BMC Med Res Methodol. 2013 Jun 4;13:72. doi: 10.1186/1471-2288-13-72 (PMC3682867; doi:10.1186/1471-2288-13-72)
Supplement: Additional file 2 — Characteristics of included studies – Qualitative aspects. [file 1471-2288-13-72-S2.pdf]

Additional File 2: Characteristics of included studies – Qualitative aspects

Study	Setting	Participants*	Topics	Key themes
Buckley 2011	Mixed methods, Ireland	FG participants recruited from general practices, with age and gender as sampling parameters. Numbers not reported.	Explored knowledge, attitudes and contexts, factors and strategies that affect attitudes. Informed development of survey.	Only key themes reported. Generally positive, contributed to “greater good” (altruistic tendencies more evident in female groups). Concerns around data security. If identifiable data then should be asked for permission or at very least informed.
Damschroder 2007	Mixed methods, USA veterans	Focus groups: 217 of the surveyed patients from Veterans Affairs (VA), who agreed to attend deliberation. Mean age of FG participants 65 years, only 5% female.	Phone survey followed by deliberation session discussing scenarios and follow-up phone survey.	Research should be "high value" for society, preferred if notes stayed within VA clinic and not sent to outside researchers, should be penalties for violations of privacy, wished to be informed about studies using their data even if they had high trust in VA, 75% not aware that data could currently be used without their permission, some concerned about stigmatizing conditions in records. Concluded that trust in VA is most powerful determinant of kind of control patients want over data.
Kass 2003	Mixed methods, USA	602 patients in one of 6 equal groups: cystic fibrosis, sickle cell disease, diabetes mellitus, HIV, breast cancer and colon cancer, recruited from outpatient clinics, ongoing research studies, disease registries and newspaper advertisement. No participant characteristics stated.	Interviews by phone or in person. The interview contained both qualitative and quantitative items.	When asked in the abstract whether they are willing to have their records used for research without their knowledge or permission, most say no. When aware that data would be anonymous and secure and they would be asked to authorise access, most believe it is a good idea.
MacKinnon 2006	Mixed methods, Canada	Reports same study as Willison 2008. 98 members of the public recruited by random digit dialling or via invitation at the end of public opinion survey.	Citizens' dialogue - 7 day-long deliberations. Had workbook in advance, detailing factual information and 3 consent approaches: scenario 1) permission each time, 2) permission not needed, but want to be informed, 3) broad consent.	Participants gave broad (one-off) consent the highest rating. Wanted more transparency about research. Strong emphasis on confidentiality and trust. Wanted more control if data was to be used for commercial purposes (shift to project-by-project consent). Lack of awareness about current safeguards.

MRC 2007	Mixed methods, UK	Qualitative workshops: 63 members of the general UK public, recruited in the street, at home and in community centres balanced by age, gender, ethnicity and SES. Qualitative interviews: 6 disabled people, people with long term illness, or their carers.	Three workshops six in-depth telephone interviews about attitudes towards using data for health research and consent.	Workshops: low understanding of medical research. Most want opt-in consent for each use but recognise this may be impractical. Data should be anonymous unless necessary. More info about research would persuade people to consent. Concern about data being used for commercial gain. Interviews with long term ill, disabled, carers: thought consent more important than general public workshops. More aware of ethic committees and safeguards. Valued individual consent more strongly, believing education about research in general was needed if researchers wanted to increase consent.
Nair 2004	Qualitative interviews, Canada	Reports same study as Willison 2003. 17 patients recruited by response to notices in waiting rooms or nomination by physician who thought they might be interested in the issues. Average age 52, 11 females.	Interviews about consent with patients whose records were being reviewed as part of a wider study.	Most unaware their data was being used despite notices in waiting room. Majority supported positive consent (n=13), but recognised the time and administrative constraints involved. All wanted to be informed of studies involving their data, believing it was courtesy to be notified. Trust in doctor, benefit to others and concern over funding from pharmaceutical and insurance companies were factors influencing their willingness to participate.
NHS IA 2002	Mixed methods, UK	Focus groups: Number of participants not reported, Interviews: 24 participants with experience of HIV/AIDS, serious genetic disorders, mental health problems, pregnancy terminations and 12 non-English speakers.<br>All participants had been referred from primary to secondary care in last year.	Shown video describing how NHS used info beforehand. Asked about use of health information and consent preferences.	Little awareness of how data used. Thought individual should be able to choose which parts of information are shared, those with mental health or pregnancy termination were least happy with routine data sharing. Data released out of NHS treatment areas should be anonymised, or consent should be requested. If anonymous data, no consent more acceptable, but some still considered it courtesy to be told. If data anonymous, secure, shared on a need to know basis with ability to withhold sensitive information, majority agree to one off written agreement between them and NHS.
Robling 2004	Qualitative focus groups, South Wales, UK	49 members of the public (plus 4 non-medical members of local community health councils) drawn systematically from electoral register in rural and urban areas.	Views on value and acceptability of 3 research scenarios: 1) GP reviews own records leading to publication, 2) transfer of names and address to external researchers, 3) transfer to external disease registry.	Concerns about access by external companies / insurance. Little knowledge of research and current safeguards. Concern about scenario 1 without consent, even though by GP, wanted opportunity to opt out. Concerns about scenario 2, as patient had to say no to release of name. Most accepting of scenario 3, anonymised and un-linked.

Shickle 2002	Mixed methods, UK	Qualitative interviews: 20 people with learning difficulties aged 18-66 recruited via day centres, 11 young women and 9 young men ages 14-17 recruited from outpatient clinics or inpatient wards. Focus groups: 13 men and 22 women from general public who indicated they would be willing to attend a focus group during other stages of the project.	Qualitative interviews and FGs, looking at various research scenarios and consent options.	Those with learning difficulties found it harder to understand concept of consent, and wanted to give the "right" answer. Young males were less concerned about information use than young females. Those with serious conditions were happier than those with little experience of NHS. Focus group participants initially concerned about data release without consent, but following discussion about practicalities they became more accepting if gaining consent was impractical.
Tracy 2004	Qualitative focus groups, Canada	28 members of the public in four groups: seniors, immigrants, urban professionals, consumer advocates recruited via telephone from relevant groups. 71% female.	FG to evaluate decision aid / tool to increase public control over how healthcare data is used and facilitate informed consent for 2' uses (HCID=healthcare information directive "to allow individuals to make informed choices about the specific types of health info they are willing to disclose (if any) for a number of specified purposes".)	Lacked knowledge about research. General mistrust of safeguards and doubts about how consent could be managed. Believed the HCID would enhance the security of health data, mixed opinions on whether it would empower individuals and increase control over data.
Willison 2003	Mixed methods, Canada	Reports same study as Nair 2004. 17 patients recruited by response to notices in waiting rooms or nomination by physician who thought they might be interested in the issues.	Interviews about consent with patients whose records were being reviewed as part of a wider study. Lead to development of a survey administered in waiting room.	Positive about research but wanted to be informed, although did not want consent to detract from reason for appointment. Unaware that data could be used without permission despite poster in waiting room, believed it was a sign of respect to be informed. Wary of drug company funding, insurance company disclosure and researchers selling data for profit to other researchers.
Willison 2009	Mixed methods, Canada	Focus groups: survey participants from one area representative of age, gender and self-reported health status who were willing to attend a focus group (number of participants not reported).	Survey followed by FGs with people with 7 stigmatizing conditions: hypertension, diabetes, depression, alcoholism, HIV, breast Ca, Lung Ca. + Ref group.	Desire for control increased when commercial profit was involved, as people felt they were being taken advantage of. Trust in doctors was key theme. Less concern about access to biological samples than income, education or occupation.

Willison 2008	Mixed methods, Canada	Reports same study as MacKinnon 2006. 98 members of the public recruited by random digit dialling or via invitation at the end of public opinion survey.	Public dialogues with workbook to read in advance, discussion about consent approaches & safeguards. Completed questionnaire before and after discussion, rating support for each of 3 scenarios: 1) permission each time, 2) permission not needed, but want to be informed, 3) broad consent.	Broad consent had greatest support in abstract, although no dominant consent opinion for scenarios. Concerned about commercial gain. Wished for greater control when profit scenario was introduced. Following dialogue opinion shifted in both directions but not significantly. Safeguards helped, but participants not aware existing safeguards. Supportive but still want control over data - no dominant consent opinion.

*For qualitative data, the number reported is the number participating unless otherwise stated
FG = focus group
MRR = medical record review
